# A deep learning based approach identifies regions more relevant than resting‐state networks to the prediction of general intelligence from resting‐state fMRI

**DOI:** 10.1002/hbm.25656

**Published:** 2021-09-29

**Authors:** Bruno Hebling Vieira, Julien Dubois, Vince D. Calhoun, Carlos Ernesto Garrido Salmon

**Affiliations:** ^1^ InBrain Lab, Departamento de Física Universidade de São Paulo Ribeirão Preto Brazil; ^2^ Tri‐Institutional Center for Translational Research in Neuroimaging and Data Science (TReNDS), Georgia State University, Georgia Institute of Technology, Emory University Atlanta Georgia USA; ^3^ Cedars‐Sinai Medical Center Los Angeles California USA; ^4^ Caltech Pasadena California USA; ^5^ The Mind Research Network Albuquerque New Mexico USA; ^6^ School of Electrical & Computer Engineering Georgia Institute of Technology Atlanta Georgia USA

**Keywords:** brain‐behavior, deep learning, fMRI, intelligence, resting‐state

## Abstract

Prediction of cognitive ability latent factors such as general intelligence from neuroimaging has elucidated questions pertaining to their neural origins. However, predicting general intelligence from functional connectivity limit hypotheses to that specific domain, being agnostic to time‐distributed features and dynamics. We used an ensemble of recurrent neural networks to circumvent this limitation, bypassing feature extraction, to predict general intelligence from resting‐state functional magnetic resonance imaging regional signals of a large sample (*n* = 873) of Human Connectome Project adult subjects. Ablating common resting‐state networks (RSNs) and measuring degradation in performance, we show that model reliance can be mostly explained by network size. Using our approach based on the temporal variance of saliencies, that is, gradients of outputs with regards to inputs, we identify a candidate set of networks that more reliably affect performance in the prediction of general intelligence than similarly sized RSNs. Our approach allows us to further test the effect of local alterations on data and the expected changes in derived metrics such as functional connectivity and instantaneous innovations.

## Graphical abstract

We predict general intelligence from RS‐fMRI timeseries using a recurrent neural network ensemble in Human Connectome Project data. We propose the selection of networks based on the variance of saliencies per ROI. Resting‐state networks (RSNs) impact on prediction can be explained by their size while with our strategy we find salient networks whose importance exceed that of RSNs.

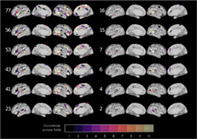

## 1 INTRODUCTION

Intelligence is comprised of a number of distinct mental abilities. According to Colom, Karama, Jung, and Haier (2010), “Reasoning, problem solving, and learning are crucial facets of human intelligence.” By means of factor analysis, a single factor was found to explain most of the empyrical positive correlation between tests (Spearman, 1904; Thurstone, 1940). Due to its generality, it was named “general intelligence,” “general factor,” or simply “g” (Jensen, 1998; Spearman, 1904). “G” was proposed as an underlying factor that dictates the overall cognitive performance of an individual and is latent to cognitive ability tests.

### 1.1 Neural bases of intelligence

The neural bases of intelligence constitute an open scientific question. Recent advances make neuroimaging a fundamental tool to answer this question. For a recent review, see Dizaji et al. (2021).

Intra cranial volume (ICV) and intelligence quotients are substantially correlated (Luders, Narr, Thompson, & Toga, 2009). This phenomenon is reproducible in both sexes and across all age groups (McDaniel, 2005). In a meta‐analysis, approximately 10% of the variance of intelligence quotients can be accounted for by differences in brain volumetry alone (McDaniel, 2005). Yet, intelligence depends on verbal, visual‐processing, information encoding and retrieval and executive tasks (Luders et al., 2009). This evokes the importance of specialized regions and networks of the brain. While ICV or gray matter volume account for much of the variation in intelligence, the remaining variance might be explained by other neurobiological factors. Potential candidates include connectivity, neuroanatomy and microstructural properties, and metabolism. Indeed, including cortical gray matter thickness estimates and white matter hyperintensity loads almost doubled the explained variance of general intelligence compared to a model only accounting for brain volume in Ritchie et al. (2015).

Previous studies were used to formulate current theories on the neural bases of intelligence. The Parieto Frontal Integration Theory (P‐FIT) proposes the existence of a single network primarily subserving human intelligence, with substantial empirical evidence derived from neuroimaging (Jung & Haier, 2007). The Network Neuroscience Theory (NNT), on the other hand, proposes that fluid and crystallized intelligence and specific skills emerge from the dynamic reorganization of networks (Barbey, 2018). This theory accommodates multiple networks and network dynamics, and relating specific network topologies to fluid and crystalized intelligence. For an overview on other competing theories, see Barbey (2018).

### 1.2 Functional magnetic resonance imaging and the biological importance of resting‐state functional connectivity

Functional magnetic resonance imaging (fMRI) allows the study of cerebral neurophysiology. Even at rest, the brain stays functionally and metabolically active. From the resting‐state‐fMRI signal, it is possible to define resting‐state functional connectivity (RSFC), the temporal coupling between signals in anatomically distinct regions of the brain (Yeo et al., 2011).

This connectivity between regions can be estimated from relatively simple measures, such as the Pearson correlation coefficients. RSFC alterations have been linked to multiple biological processes, such as brain disorders, aging, and cognition. More importantly, intelligence has also been found to correlate with RSFC and graph theoretical measures, such as local efficiency (Pamplona, Santos Neto, Rosset, Rogers, & Salmon, 2015). More recently, fMRI‐derived data has been used to perform predictive analyses at the individual‐level (Sui, Liu, Lee, Zhang, & Calhoun, 2020).

Other than estimating temporal coupling between time series, it is possible to define spatially independent components of blood‐oxygen‐level‐dependent (BOLD) contrast fluctuations using data‐driven blind signal separation techniques such as independent component analysis (ICA; Calhoun, Adali, Pearlson, & Pekar, 2001; Calhoun & Adali, 2012). In addition to structured noise components, neural components have been robustly identified with ICA as resting‐state networks (RSNs), with large empirical evidence corroborating their existence. ICA‐derived RSN topologies found validation with other techniques, such as magnetoencephalography (Brookes et al., 2011) or community detection with discrete regions (Ito et al., 2017). These RSNs are often linked to known cognitive, sensory, or motor processes. The connectivity or topology of RSNs is often used to deduce the cognitive effect of the alterations observed.

### 1.3 Predicting “g” from resting‐state functional imaging

Individualized intelligence estimation from RSFC based on machine learning is already in practice (Dubois, Galdi, Paul, & Adolphs, 2018; Finn et al., 2015). The most successful approaches to the prediction of intelligence are often based on linear modeling with univariate feature filtering. Even though it is not theoretically a required step, it was employed by most of the successful approaches (Dizaji et al., 2021). Under this type of model, when a feature is discarded it can no longer contribute with the predictions, no matter how much it is altered. This implausibility, compounded with our prior knowledge of how the brain works, motivate us to search for other approaches.

The brain is a complex entity with complex dynamics. The interplay between these dynamics and biological processes such as intelligence can be presumed to be complex as well. The use of aggregate measures, for example, RSFC, can be used to inform highly predictive models with a window for direct interpretability. At the same time, that choice limits the hypotheses about the data. Deep learning can be used to learn predictive representations of data with automatic feature extraction (Abrol et al., 2021). Employing deep learning to learn about the biological bases of “g” opens up new possibilities for interpretation of results, removing the bias due to the choice of features. Human‐level interpretability is more challenging due to the increased complexity of the models. A few strategies exist, however, that allow for insights to be extracted from modeling. We explore two such strategies, namely feature ablation and saliency (Molnar, 2019).

The effectiveness of deep learning in predictive analyses in cognitive neuroscience over “classical” machine‐learning methods, such as linear, kernel‐based, and tree‐based models, has been a topic of recent debate. While some studies show comparable performance between both paradigms (He et al., 2020), deep learning methods have been shown to outperform classic machine learning as well (Abrol et al., 2020). Deep learning models are able to explore existing nonlinearities in neuroimaging data and automatically extract informative features (Abrol et al., 2021). This allows deep models to often surpass traditional machine learning models in performance. Since highly flexible models are more prone to variance, we control their variance via ensembling, that is, averaging predictions networks trained independently on the same data.

In this work, we aim to demonstrate that learning from lower level data, that is, timeseries instead of RSFC, can bring further insights into the question of the neuronal bases on intelligence. This type of data requires specialized models, and we opt to use an ensemble of recurrent neural networks (RNNs) for this task. We performed the prediction of “g” from fMRI timeseries obtained from a discrete cortical parcellation. To the best of authors' knowledge, this is the first work to perform this type of analysis. We then interpret the model predictions on unseen data performing ablation and saliency studies. We show that the ablation of single anatomical regions does not degrade performance and that the degradation in performance when ablating RSNs is not greater than when ablating random sets of regions with equal size. We calculate model reliance based on temporal variance of saliencies and show that, when ablating sets of regions ranked by this measure, significant degradation of performance ensues compared with the random sets. We then propagate saliencies to derivative measures and derive neuroscientific insights from the nature of observed saliencies.

## 2 METHODS

### 2.1 Data and preprocessing

Original data were provided by the Human Connectome Project (HCP; Essen et al., 2013). Preprocessed and behavior data were provided by Dubois et al. (2018). Briefly, test scores from 1,206 subjects were obtained. Tests include seven tasks from the NIH Toolbox for Assessment of Neurological and Behavioral function (dimensional change card sort; flanker inhibitory control and attention; list sorting working memory; picture sequence memory; picture vocabulary; pattern comparison processing speed; oral reading recognition) and three from the Penn Computerized Neurocognitive Battery (Penn progressive matrices; Penn word memory test; variable short Penn line orientation). Twenty‐three subjects with missing or incomplete test scores were excluded (*n* = 1,183). Two subjects that scored 26 or less in the Mini Mental State Examination (MMSE) were also excluded (*n* = 1,181). These subjects were available for factor analysis. Subjects that completed four imaging sessions (*n* = 998) were further filtered by excluding 114 subjects with excessive in‐scanner head movement (*n* = 884). Preprocessing included *z*‐standardization, removal of temporal drifts from white‐matter and CSF signals, regression of mean white‐matter and CSF signals from gray matter, regression of six realignment parameters and their first derivatives, low‐pass filtering with a Gaussian with a *SD* of 720 ms, removal of temporal drifts from the resulting gray matter signal, and finally global‐signal regression. Temporal drift removal was based on third‐degree Legendre polynomials. See Dubois et al. (2018) for details. We further removed 11 subjects with less than 1,200 timepoints per session, achieving a final set of 873 subjects with complete imaging and behavioral data.

Maximum likelihood factor analysis was performed in Dubois et al. (2018) including one general factor and four lower‐order factors. The Schmid–Leiman transformation is used to derive loadings for the general factor. Individual scores are obtained by the Thurstone regression method.

We applied band‐pass filtering (BPF) at [0.008 Hz, 0.09 Hz] to the ROI timeseries. This frequency band corresponds to the expected slow BOLD fluctuations, increasing signal specificity. This is due to the hemodynamic response function attenuation (Sun, Miller, & D'Esposito, 2004), typically up to 0.15 Hz. The major drawback of BPF is that it possibly reduces sensitivity, since higher frequencies might still pertain to the task. Data were then decimated. This entails truncating the spectra at 0.09 Hz and then transforming data back to the time domain. We obtained 155 timepoints per session after decimation. Dividing the session length of 864 s by 155, this leads to a virtual TR of 5.57 s. This additional procedure reduces the data dimensionality without loss of information and removes the long‐range temporal autocorrelation induced by BPF. Individual sessions were kept separate.

### 2.2 Neural network architecture

We opted to build upon a simple RNN based on the long‐short term memory (LSTM) (Hochreiter & Schmidhuber, 1997) module. The LSTM captures both short and long‐range information in sequences. It has been shown to work efficiently empirically, including applications in neuroimaging data (Dvornek, Ventola, Pelphrey, & Duncan, 2017).

To capture complex dynamics and also to better distribute gradients in the network, we used the BiLSTM module (Graves & Schmidhuber, 2005). The BiLSTM consists of two LSTM layers applied in parallel to time‐distributed data. One of the layers transverse the data forward in time, while the other does the same backward. The activations of both are then combined at each timestep by adding both, in our case.

**FIGURE 1:**
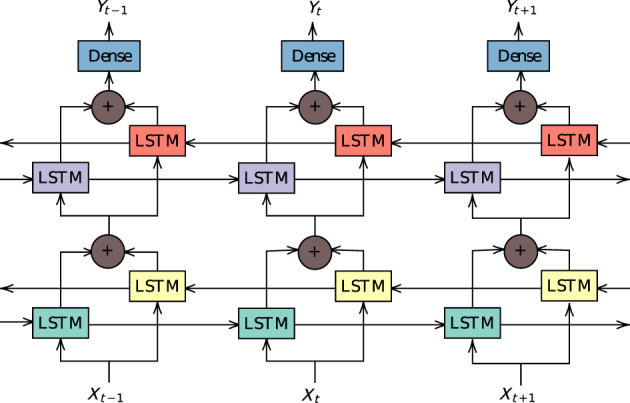
Illustrated architecture of two‐layer bidirectional recurrent neural networks. Matching colors signify shared weights. The inputs $X_{t}$ are 360‐dimensional vectors of pseudo‐activity in each ROI. Every LSTM module outputs a 256‐dimensional vector. The outputs $Y_{y}$ are scalar and are subsequently mean‐pooled. Parameters are optimized to predict “g”

Our architecture, represented in Figure 1 consists of two BiLSTM modules and a linear layer with identity activation. The first BiLSTM has 360 inputs (corresponding to each ROI defined in the atlas) and 256 outputs, while the second has 256 inputs and outputs. The activations of the latter are mean‐aggregated, resulting in a 256‐dimensional vector per timeseries. This representation is fed to the linear layer, resulting a single scalar output. Parameters are optimized so that the average value of these scalars best predict “g.” We implemented our architecture in the framework Flux (Innes, 2018), written completely in Julia (Bezanson, Edelman, Karpinski, & Shah, 2017). It has 2,316,545 learnable parameters in total, 632,320 and 525,824 in each LSTM in the first layer and second layers, respectively, and 257 in the affine layer. Based on this architecture, we trained an ensemble of 50 networks trained independently with the same data configuration. Final prediction is obtained by averaging the prediction of each member of the ensemble. For comparison, the convolutional architecture for image classification AlexNet (Krizhevsky, Sutskever, & Hinton, 2012) has almost 61 million trainable parameters.

### 2.3 Training

During training, we decorrelated general intelligence from gender, age, brain volume, movement from each resting‐state session, and reconstruction algorithm version, in accordance to Dubois et al. (2018). This procedure eliminates possible contributions of no interest from “g,” augmenting specificity. General intelligence was then rescaled to unit variance and centered to zero mean.

We trained our architecture with backpropagation through time (BPTT; Williams & Peng, 1990). In our learning scheme, each LSTM layer is fed a single batch timepoint, producing its respective output and LSTM cell state. In the next timestep, the layer is again fed the batch input and takes the previous cell state to produce its output. This is repeated until the end of the sequence, when we calculate gradients for the backward pass on the objective function and update the parameters of the network accordingly.

We used mean‐pooling on the outputs of the forward pass during training. Each sequence had up to 20 timesteps removed during each batch optimization, both at the start and also at the end, providing some variability to the training scheme. The four sessions of resting‐state timecourses were fed separately and in random order for the forward pass of the network and were effectively treated as separate training samples. This subtle difference stimulates the network to obtain the best possible estimate from each session instead of vying to optimize the average between sessions.

We trained the architecture for 50 epochs using the “ADAM” optimization algorithm (Kingma & Ba, 2014) to perform update iterations. The learning rate was set to 0.0005. We also regularized gradient descent by employing Weight Decay (Krogh & Hertz, 1992) with a decay constant equal to 0.0005. Due to the adaptive nature of “ADAM,” Weight Decay, and ${ℒ}_{2}$ regularization do not coincide. We used decoupled Weight Decay (Loshchilov & Hutter, 2019) to overcome that limitation.

### 2.4 Validation

We performed 10‐fold stratified cross‐validation. We stratified families based on the terciles of family‐averaged “g” and family size, with groups of families with 1, 2, and 3 or more members. This stratification was conducted in order to make validation folds more homogeneous. We computed folds from each stratum of families, and then recombined these folds across strata to ensure a homogenous distribution of “g” across folds. The number of families in each stratum is shown in Table 1.

**TABLE 1:** Stratification of families into average “g” terciles and family size

		Family size		
		1 (*n* = 115)	2 (*n* = 156)	3+ (*n* = 138)
**“g” terciles**	T1 (*n* = 135)	53	47	35
	T2 (*n* = 135)	25	54	56
	T3 (*n* = 139)	37	55	47

The forward pass during validation was virtually exactly the same as in training. The only exception is that outputs were generated by mean‐aggregating outputs from all timepoints within a session, and then averaged across sessions. The general intelligence of validation data was transformed in accordance to the transformation obtained for training data in each fold. This prevents leakage of validation data into training data. The procedure includes decorrelation of confounder variables, rescaling and centering.

### 2.5 Performance‐evaluation, comparison, and model exploration

We employed three performance metrics. The MSE (mean squared error) was used during training. To evaluate performance on validation data we computed the coefficient of determination $R^{2}=1-\mathrm{MSE}\left(y,\hat{y}\right)/\mathrm{Var}\left(y\right)$. We also report the squared correlation coefficient $\rho^{2}=\mathrm{Cor}\left(y,\hat{y}\right)$, to allow for comparison with other works that used this metric exclusively. y and $\hat{y}$ are vectors of true values and predictions, respectively.

We implemented elastic‐net regression to serve as an alternative model for comparison, predicting “g” from the RSFC, averaged between sessions. See Dubois et al. (2018) for more details. Input data were filtered in univariate fashion at each fold. This procedure is based on the correlation between each input feature and “g” for training data. We set the parameter balancing the LASSO and ridge penalties to α=.05. This leads to almost pure ridge regularization. The parameter λ that controls the tradeoff between the loss function and the penalization was optimized in an inner three‐fold cross‐validation. This inner optimization was based solely on training data at each outer fold. λ was optimized based on a grid with 50 values. This whole model was validated on the same fold configuration mentioned previously in Section 2.4.

To further understand how the ensemble model behaves, we performed two model‐agnostic exploration strategies: ablation and saliency (Molnar, 2019). This allows us to understand what information the model relies on to reach the performance we assessed in cross‐validation. Both techniques were applied to validation data.

### 2.6 Model exploration: ablation study on validation sets

We performed an ablation study on the trained model. Ablation consists in removing information from input and assessing the respective degradation in performance. We ablated anatomically‐defined atlas regions and entire functional networks. This procedure should be able to discern model reliance on individual features and combinations of features. For the networks, we used the network definition established by Ito et al. (2017), which is based on the same MMP cortical atlas we employed.

To account for the fact that networks have different sizes, we compared the statistics obtained with a distribution of statistics. This distribution was obtained from resampling random networks with matching sizes to the one being tested. This way, we can more certainly state that a change in performance after ablation is not simply due to the removal of nodes. The procedure consists of, given a network with M nodes, selecting M nodes at random to be ablated. The statistics associated with the performance are stored and the procedure is repeated for 300 iterations, in our case. We extracted the paired T‐statistics, comparing the average performance across the 10 folds with and without ablation, with a null‐hypothesis of zero difference. We then define a *p*‐value for each hypothesis tested as the proportion of resampled statistics from the pool that are more extreme than the statistic measured empirically. We used p<.05 as a significance threshold throughout, after correcting for multiple comparisons at the analysis level with the false discovery rate controlling procedure described in Yekutieli and Benjamini (1999).

### 2.7 Model exploration: saliency analysis on validation sets

Saliency measures the degree that a measure is influenced locally by perturbations. It is often defined as the partial derivative of the outputs of a model on its inputs. We chose to study the saliency of the trained models to understand what features of brain resting‐state activity are related to “g.” Since our model is fully differentiable, we can easily compute this measure. For each fold, we obtained the saliencies $\partial_{X}N\left(X\right)$ based on validation, that is, unseen, data. The saliencies have the same dimensionality of input data since our output is scalar.

Due to efficiency reasons, mean centering and unit scaling and temporal filtering are often performed outside cross‐validation per timeseries. When analyzing saliencies, however, we must take into account this normalization, as it is a constituent part of our model. Otherwise, we might observe, for example, a non‐null gradient at frequency zero, which is not possible under our model, as it assumes all data was standardized. We used the chain rule to propagate derivatives to standardized filtered data $\partial_{S}N\left(X\right)=\partial_{S}X\cdot \partial_{X}N\left(X\right)$. For more details, see Appendix A. In practice, however, the procedure is performed automatically using automatic differentiation.

Since we take into account standardization, average saliency of a region is zero across time, by design. We instead summarize reliance on regional timeseries by the temporal variance of saliency, defined, for a region timeseries $x_{i}$, as $\sum_{t}^{T}{\left(\partial_{x_{\mathrm{it}}}N\left(X\right)\right)}^{2}/\left(T-1\right)$.

### 2.8 Ablating a network defined by the regions with highest saliency

Regions with high average squared saliency should be approximately the regions the model relies on more for local changes in the estimate of “g.” Thus, it makes sense to use that as a proxy of model reliance, per region. Using the temporal variance of saliency per region, we define 12 networks that have each the size of the 14 RSNs defined in Ito et al. (2017). For each of these “saliency‐based networks” we perform the ablation and permutation study described in Section 2.6. To avoid circular analysis, that is, “double‐dipping,” we cross‐validate this procedure. We employ saliencies from training data to define which regions are ablated in validation data.

### 2.9 Functional connectivity saliency

Apart from the original data, we also explored saliencies on functional connectivity. Given that saliencies denote a change in input data, we can propagate this to functional connectivity. Since our saliencies preserve the mean and variance of data, we can plug them directly into the definition of functional connectivity. Equation (1) shows the update operator for X and, likewise, $C=M^{-1}X^{T}X$.

(1) $$\left\{\begin{array}{l} X'≔X+\eta \frac{\partial N\left(X\right)}{\partial X}\\ C'≔\frac{1}{M}X^{T}X+\frac{\eta }{M}\left({\frac{\partial N\left(X\right)}{\partial X}}^{T}X+X^{T}\frac{\partial N\left(X\right)}{\partial X}\right)+\frac{\eta^{2}}{M}\left({\frac{\partial N\left(X\right)}{\partial X}}^{T}\frac{\partial N\left(X\right)}{\partial X}\right)\\ \delta C\approx \frac{\eta }{M}\left({\frac{\partial N\left(X\right)}{\partial X}}^{T}X+X^{T}\frac{\partial N\left(X\right)}{\partial X}\right) \end{array}\right.$$

In Equation (1), since η≪1, the third term of C' is, again, very small. Therefore, we focus on the second term, δC. This local change in connectivity is proportional to the covariance between the data and the saliencies. δC is the associated alteration in connectivity from changing activity to increasing the “g” estimates in the model.

### 2.10 Propagating saliency to decorrelated inputs through the zero‐phase component analysis

When investigating saliency on the original data, one can incorporate possible data generating processes. We assume that RSFC generates the data acting on instantaneous innovations in each region. This can be described as X=ZW. X is the centered and unit‐scaled data, where each column is a region and each row is a timepoint, Z represents the (uncorrelated) innovations and has the same size of X and W is a square dewhitening matrix, thus $\left(n-k\right)C=X^{T}X={\left(\mathrm{ZW}\right)}^{T}\left(\mathrm{ZW}\right)=W^{T}W$, because $Z^{T}Z=I$. W is then proportional to the square‐root of the correlation matrix, $W={\left(n-k\right)}^{1/2}C^{1/2}$, thus $Z={\left(n-k\right)}^{-1/2}{\mathrm{XC}}^{-1/2}$. Taking the singular value decomposition (SVD) of X results in $X={\mathrm{USV}}^{T}$, then the inverse square root of the correlation is simply ${\left(n-k\right)}^{-1/2}C^{-1/2}={\mathrm{VS}}^{-1}V^{T}=W^{-1}$. Therefore, we can estimate $Z={\mathrm{XW}}^{-1}={\mathrm{XVS}}^{-1}V^{T}=U\left(S\left(V^{T}V\right)S^{-1}\right)V^{T}={\mathrm{UV}}^{T}$.

This procedure, called zero‐phase components analysis (ZCA), was described in Krizhevsky (2009). It results in decorrelated variables that best correspond to the original ones. Or, in other words, each column of Z is proportional to the residuals of a least‐squares linear regression that predicts the timeseries of a region based on all other regions. Propagating saliencies to Z is then a matter of propagating saliencies through the SVD. For details, refer to Appendix B.

We propagate saliencies in all subjects, sessions, and folds to Z using automatic differentiation. Studying dZ allows us to understand the model reliance on regional timeseries beyond RSFC. Regions with high temporal variance in dZ might have low temporal variance in dX because their activity is propagated to the rest of the network due to connectivity. Conversely, regions with high temporal variance in dX might have low temporal variance in dZ, because the model relies on them due to their shared information with other, more informative, regions. Note that, $W^{2}\left(n-k\right)={\mathrm{VS}}^{2}V^{T}=C$. If we applied the same SVD differential identities presented here to C, the same result in Equation (1) would be obtained.

## 3 RESULTS

### 3.1 Confounder variables explain substantial variance of “g”

As part of our framework, we predict “g” in validation data across folds using the coefficients estimated from training data and perform additional analyses on the residuals of this model. We obtain $\rho_{\text{mean}}^{2}=0.138$, $\rho_{\text{stderr}}^{2}=0.0279$ across folds. These confounders include gender, age, brain volume, movement from each resting‐state session, and reconstruction algorithm version. This result is in line with previous works (Dubois et al., 2018).

### 3.2 Penalized linear modeling predicts “g” from RSFC

To be able to compare results we implemented the modeling approach presented in Dubois et al. (2018) using our validation scheme. Using this approach, we obtained $R_{\text{mean}}^{2}=0.170$, $R_{\text{stderr}}^{2}=0.0264$, and $\rho_{\text{mean}}^{2}=0.178$, $\rho_{\text{stderr}}^{2}=0.0242$ across folds.

### 3.3 RS‐fMRI timeseries predict “g”

Using our ensemble, we obtained $R_{\text{mean}}^{2}=0.184$, $R_{\text{stderr}}^{2}=0.0149$ and $\rho_{\text{mean}}^{2}=0.194$, $\rho_{\text{stderr}}^{2}=0.0170$ across folds on validation data.

If we were to add the penalized linear model to the ensemble we obtain $R_{\text{mean}}^{2}=0.197$, $R_{\text{stderr}}^{2}=0.0184$ and $\rho_{\text{mean}}^{2}=0.206$, $\rho_{\text{stderr}}^{2}=0.0203$. This small increase in cross‐validated performance is due to the high shared variance between predictions, $\rho_{\text{mean}}^{2}=0.622$, $\rho_{\text{stderr}}^{2}=0.0193$. For comparative purposes, analogous results are obtained when not removing the effect of confounder variables. The performance of the baseline model and the deep ensemble equal $R_{\text{mean}}^{2}=0.183$, $R_{\text{stderr}}^{2}=0.0286$, and $R_{\text{mean}}^{2}=0.204$, $R_{\text{stderr}}^{2}=0.0178$, respectively. Both models sharing $\rho_{\text{mean}}^{2}=0.621$, $\rho_{\text{stderr}}^{2}=0.0609$ variance.

The impact of ensemble size on validation performance is shown in fig:ensemblesizeperformance.

### 3.4 Ablation of single regions does not affect performance

We show in Figure 3 how removing one region at a time from the validation data affects performance. Colors represent each network assignment defined in Ito et al. (2017).

### 3.5 Ablation of RSNs alter performance; their size explains this effect

We also performed an ablation study deleting one RSN at a time. This is shown in Figure 4. We assessed significance with the paired *T*‐test. We compared the average performance across folds in the baseline with the average performance after ablating each RSN. Results were corrected for multiple comparisons using the procedure described in Yekutieli and Benjamini (1999).

However, resampling the distribution of *t*‐statistics with random, equally‐sized networks, we did not observe any significant effect in any performance metric. This implies that the alterations in performance observed in Figure 4 can be explained by the size of RSNs alone.

### 3.6 Peak regional saliency variance describes a network that when ablated significantly deteriorates model performance

Figure 5 shows 12 networks, each matching in size the 14 RSNs defined in Ito et al. (2017). These networks are defined from regions that exhibit the largest temporal variance of saliencies across subjects and sessions. Since they are obtained from training data occurrence differs across folds. Only 12 networks are defined because two networks have two regions (PCC and PREM2) and two networks have 15 regions (AUD1 and HIPP). The bigger networks contain all the regions in the smaller ones, that is, smaller networks are subsets of bigger ones.

When comparing the degradation in performance measured by $R^{2}$, after correcting for multiple comparisons, most saliency based networks degraded performance significantly more than equally sized random networks. This is shown in Figure 6.

### 3.7 Increasing intelligence estimates accompany expected alterations in functional connectivity

Figure 7 shows the expected value of the change in connectivity δC that accompanies increasing “g.” As in Ferreira et al. (2016) we choose to categorize saliencies with regards to their average magnitude and the direction of change to ease visualization. Only the highest 5% effects, defined as the average saliencies divided by the respective *SD*, are shown. No correlation was found between the values of average δC and average C.

### 3.8 Saliency on whitened timeseries *Z* demonstrates the weight of RSFC on model reliance

Figure 8 shows the average temporal variance of saliency obtained from whitened data, based on ZCA. Regional contributions are lessened due to the removal of functional connectivity, an expected result.

## 4 DISCUSSION AND CONCLUSION

Performance of the ensemble is on par with reported in Dubois et al. (2018). Here we employ more restrictive data, including decimation, which should increase specificity at the cost of sensitivity. Training the ensemble with leave‐one‐family‐out cross‐validation would increase computational costs prohibitively. We thus employ 10‐fold CV, which entails less training data in each resample, but displays less variance than leave‐one‐out cross‐validation at the same time (Kohavi & Edu, 1993). Both effects can be noted by the slightly deteriorated performance we observe when validating the penalized linear model. In our data, we retrieve $R_{10-\text{foldCV}}^{2}=0.170$, compared to $R_{\text{LOFO}}^{2}=0.206$ reported in Dubois et al. (2018). Increasing the training set size would increase performance for both the ensemble and the penalized linear model.

Having said that, a modest 8% increase in performance is noted when using our model. This suggests we successfully captured the effects of interest. Given the increased complexity of our model it becomes clear we are possibly reaching the point of capturing most information available in the data. Future works could explore data sampled at other spatial granularities, culminating in using the voxel timeseries themselves. Other timescales can be explored, either fast changing neural oscillations afforded by techniques such as MEG and EEG, or slow changes in activity patterns with longitudinal data.

**FIGURE 2:**
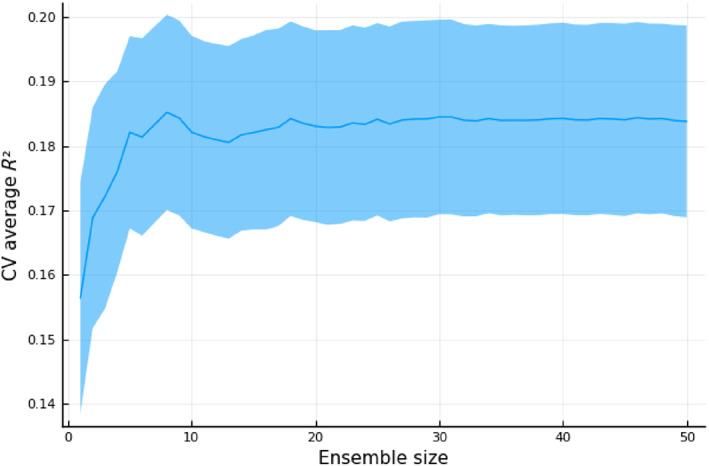
Effect of ensemble size on cross‐validated average *R* ^2^. Ribbon shows the *SE*. Ensemble members were added consecutively in the same order of training

We chose to ensemble RNNs to control their variance. Figure 2 demonstrates that we reach a plateau on performance long before summing 50 models in our ensemble. Roughly, 10 models achieve peak performance in our scenario.

We choose to assess importance on validation data. An equally valid choice would have been studying training data prior to learning. Dubois et al. (2018) applies this approach to study RSNs. For a discussion, see Molnar (2019).

**FIGURE 3:**
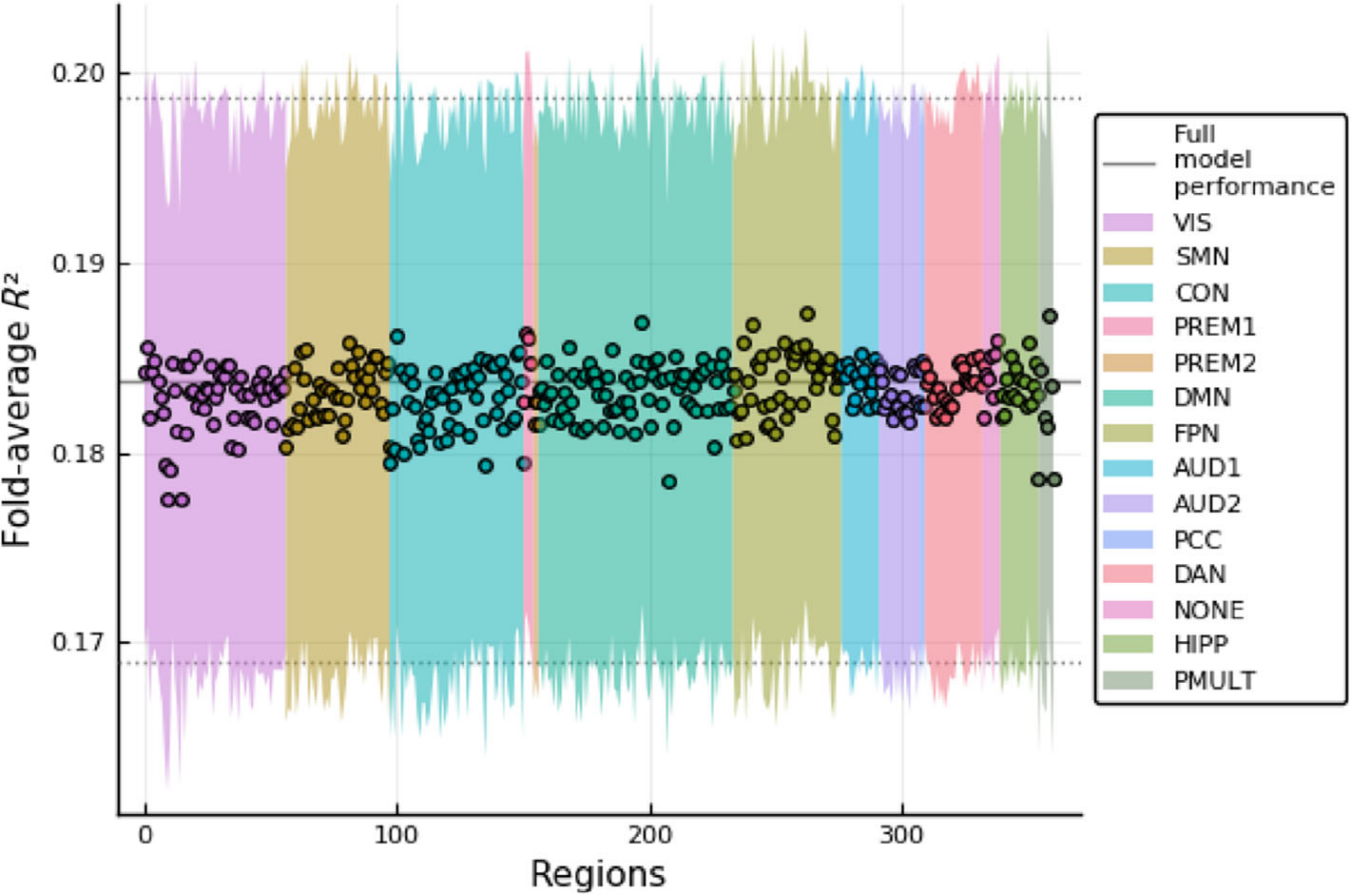
Effect of regional ablation on cross‐validated average *R* ^2^. Ribbon shows the standard error. The black line represents the performance measured when using all data. Colors represent each resting state network assignment defined in Ito et al. (2017)

As expected, Figure 3 shows that the removal of no single region is sufficient to substantially disrupt model performance on validation data. This suggests that, as other works have shown, intelligence is distributed across different regions in the brain (Dubois et al., 2018). This result is compatible with the initial premises of current theories, such as P‐FIT and NNT.

**FIGURE 4:**
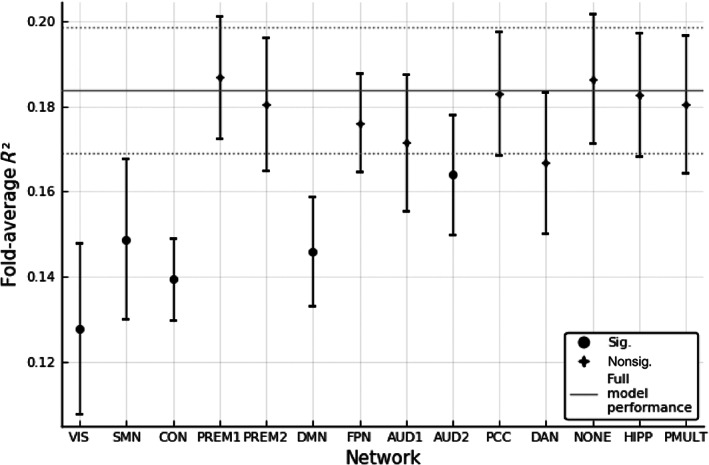
Effect of network ablation on cross‐validated average *R* ^2^. Error bars represent the *SE*. The black line represents the performance measured when using all data. Significance was assessed with the paired *T*‐test, comparing performance in the baseline with the ablation of each network across folds. The Benjamini–Hochberg procedure was employed to correct for multiple comparisons

When looking into RSNs, as shown in Figure 4, removing one of the visual, somato‐motor, cingulo‐opercular network (CON), default mode network (DMN), or auditory networks significantly lowers performance on the validation set. These networks have been reported before as important networks for intelligence. However, some of these networks are also among the biggest in the atlas in Ito et al. (2017). This led us to investigate a direct effect of the amount of information being removed. We found no significant difference in the decrease in performance to the one obtained with resampled random networks of the same size. This means that we have no evidence that the ablation of RSNs reveals specific reliance on these networks for prediction. Rather, the size of RSNs explains this effect.

As exposed in Section 2, by design saliencies sum to zero and their variance is such that, locally, it cancels their covariance with data. This is important for their validity regarding the full model. It also precludes us from exploring such simple measures as average saliency.

Results point to the fact that saliency is heterogeneous across the cortex. Additionally, we did not detect bilateral patterns. This could be due to redundancies in information between homotopic regions. Ventral visual areas display low saliency overall. Wernicke's and Broca's areas are highlighted with high saliency temporal variance.

The role of DMN deactivation in cognition has been studied elsewhere. See Anticevic et al. (2012) for a review.

To define a measure of regional reliance, we employ the temporal variance of saliencies. Saliencies can be interpreted as the change in data that leads to an increase in the output. Thus, regions with high temporal variance of saliencies are also the ones being most altered.

**FIGURE 5:**
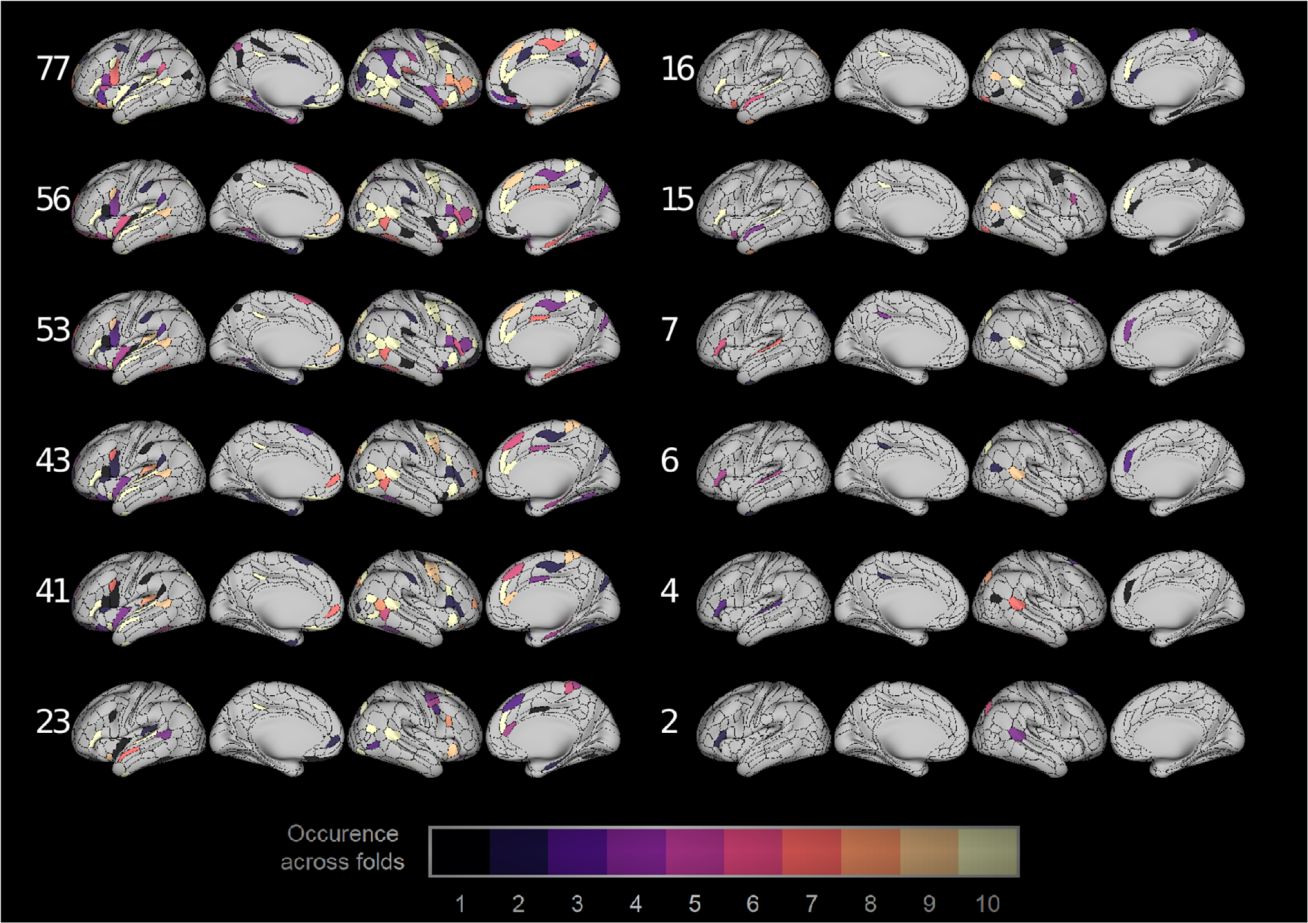
Regions with top saliency temporal variance in training data across folds. Occurrence goes from 0 to 10. This selection is used to ablate regions in validation data. The number of regions encompassed by the “networks” is shown in white next to each row

**FIGURE 6:**
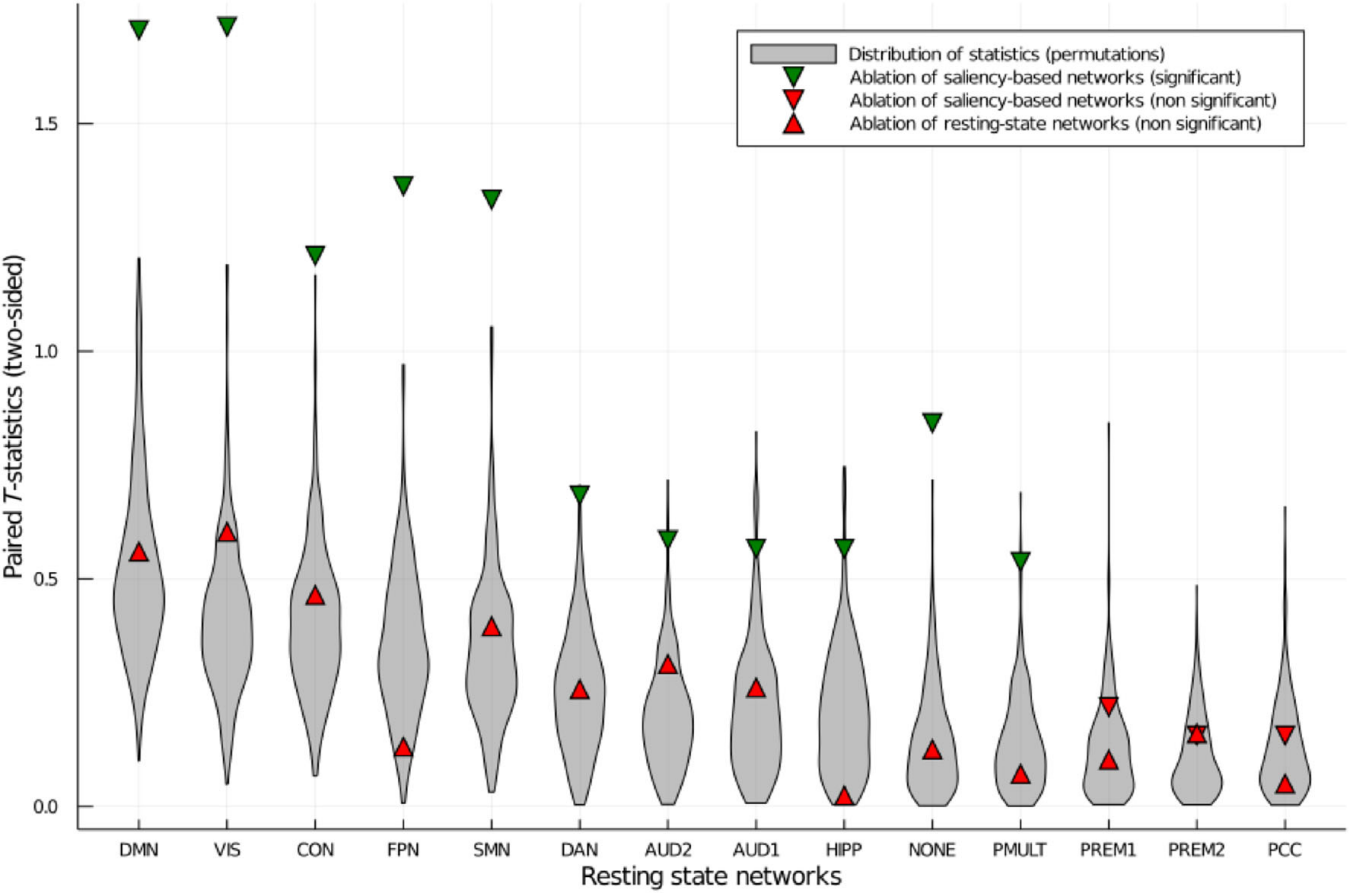
Comparison of degradation in performance by ablation of networks. Paired *T*‐test statistic comparing ablated results with original performance across folds. The gray ribbon represents a randomization‐based distribution using random networks with the same number of nodes of corresponding RSNs. Downward‐pointing triangles represent the test statistics obtained when ablating networks defined in Figure 5. Upward‐pointing triangles represent the test statistics obtained when ablating RSNs. Significant effects when compared with the randomization‐based distribution are shown in green. Nonsignificant results are shown in red. RSNs are ordered largest to smallest, from left to right

Ablation of the “networks” shown in Figure 5 demonstrated significant reductions in performance when compared with resampled random networks, shown in Figure 6. These networks were obtained by keeping only the regions with highest average saliency temporal variance in cross‐validated fashion. Anatomically, the selected regions cover many parts of the brain. Many regions in the frontal lobe are included, including orbitofrontal, ventrolateral, and dorsolateral prefrontal and the pregenual anterior cingulate. Ventral occipital and ventral temporal areas are present in both hemispheres, as well as ventral anterior insula regions and inferior parietal regions. Dorsomedial frontal and posterior temporal regions are selected in the right hemisphere but lacking in the left. In the left, superior temporal regions are featured prominently. Very few regions have their homotopic counterpart featured.

These regions populate several RSNs. All regions of the DMN are included with the exception of the angular gyrii. Fronto‐parietal network (FPN), ventral visual and ventral and dorsal attention, right medial somatomator regions, and left auditory regions are contemplated. Several regions, however, lie on the frontier between major networks. We might speculate that the individual extent of RSNs could contribute to the prediction of “g.”

The selections of networks in Figure 5 include prominent P‐FIT areas. Notably, prefrontal, parietal, and temporal associative areas are included. However, bilateral visual associative regions are missing. We also do not verify any prominence of the left hemisphere over the right one, as theorized in the P‐FIT. This can be an artifact of the number of regions selected or the interindividual variability in functional localization. Of particular interest is the confounding effect of information shared through connectivity. This effect will be further discussed below.

**FIGURE 7:**
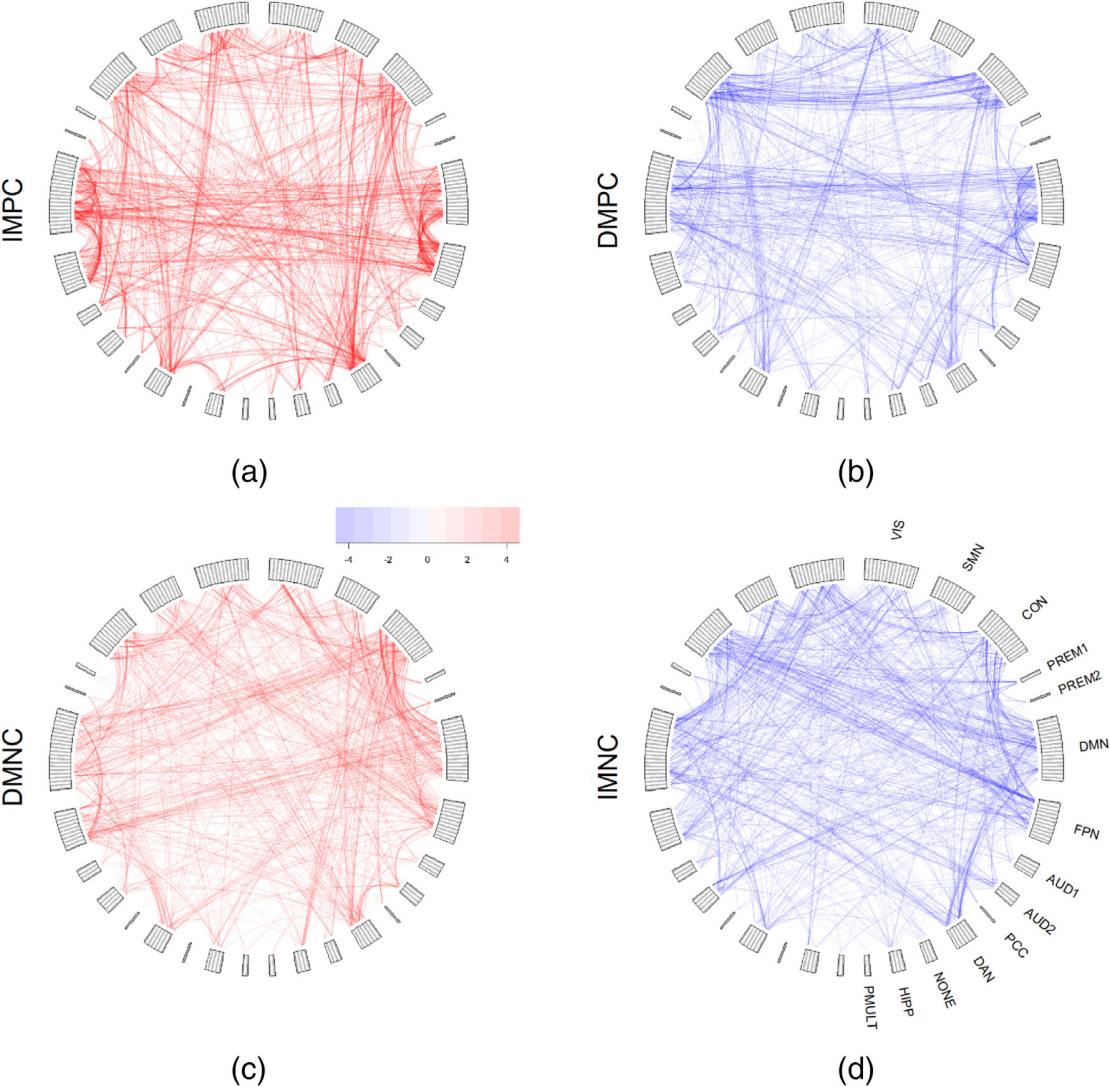
Average derivative of functional connectivity in the direction of increasing “g,” standardized by the *SD* across samples. (a) Increased magnitude of positive correlations (IMPC). (b) Decreased magnitude of positive correlations (DMPC). (c) Decreased magnitude of negative correlations (DMNC). (d) Increased magnitude of negative correlations (IMNC). Highest 5% effects are shown. Opacity increases with effect magnitude

Comparing the highly modularized functional connectivity between RSNs with the average functional connectivity saliency shown in Figure 7, it is clear that the latter does not share the same modules as the first. This implies no simple alignment with intra‐ or internetwork connectivity. Instead, we observe that a small number of regions exhibit marked saliency to connectivity with specific networks. These are easily spotted as blue or orange lines in Figure 7, respectively average increasing and decreasing of functional connectivity with increasing “g.” In the case of increasing connectivity with a whole network, this would indicate that a region is more integrated with that network with increasing “g.” Likewise with decreasing functional connectivity, said region would be more segregated from that specific network. The CON exhibits marked loss of intranetwork connectivity with increasing “g.” Some visual, DMN, and FPN regions show uniform decreasing connectivity to it as well.

Comparing the top row with the bottom row in Figure 7, it becomes evident that, among the 5% highest effects, positive correlations are more prevalent than negative correlations. Positive correlations are expected to occur in intranetwork connections, while negative correlations occur in internetwork connections. In Figure 7a, DMN and FPN regions show evidence of increased coupling in the direction of increasing “g.” In Figure 7b, on the other hand, other DMN and FPN regions become decoupled. In the latter, CON intranetwork connections become weaker in the direction of increasing “g.” In the case of negative correlation shown in Figure 7c,d, which occur mostly between RSNs, bilateral CON, DMN, and FPN connectivities are highlighted. The role of networks such as the CON, DMN, and FPN RSNs in intelligence was previously studied (Dubois et al., 2018; Hearne, Mattingley, & Cocchi, 2016). These three networks, in particular, are coupled: the CON is associated with switching levels of activity between DMN and FPN (Sridharan, Levitin, & Menon, 2008).

**FIGURE 8:**
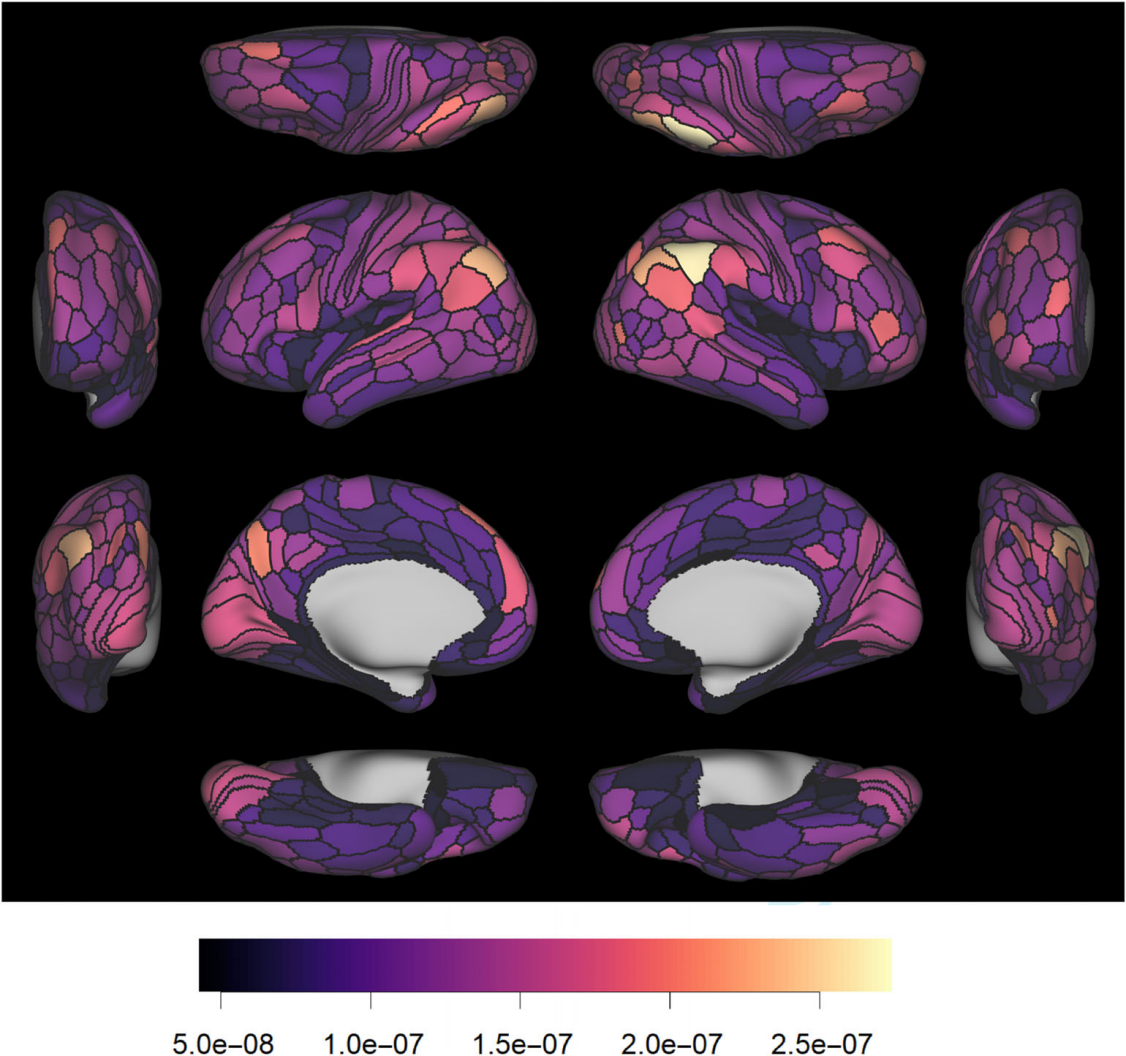
Average temporal variance on saliency calculated over whitened data, that is, multivariate uncorrelated innovations

After disambiguating the role of spontaneous activity, that is, temporal innovations, in the saliencies, other regions emerge in Figure 8. In special, the inferior parietal lobules have high saliency in this regard, including the angular gyri and the supramarginal gyri. These two regions are involved in language streams and several higher order cognitive functions. They are directly connected to the frontal lobe by the superior longitudinal fasciculus, including dorsolateral prefrontal cortices. P‐FIT places high importance to this area of the cortex, especially the left angular gyrus (Jung & Haier, 2007). Again, the left primary auditory cortex has high saliency, the sole primary sensorial region to be so, which is also accommodated within P‐FIT.

On the other hand, areas such as the insulas, parahippocampal and entorhinal cortices, and the subgenual area are less pronounced in Figure 8. These areas are absent in P‐FIT (Jung & Haier, 2007), further corroborating our results.

We can hypothesize that regions that were highlighted in Figure 5 but not in Figure 8 could attain high saliency due to their connectivity. The converse is also true, where regions highlighted in Figure 8 but not in Figure 5, under this hypothesis, would be so due to their connectivity.

The smallest “networks” containing two or four regions do not display significance in Figure 6. Low concordance was obtained across folds for these, as can be seen in Figure 5. This adds to the point that intelligence cannot be attributed to single regions. It is very likely that the choice of “networks” that degrade performance more than random, equally‐sized, ones is not unique. While other “networks” can possibly attain the same effect, the point is that RSNs are not, in fact, specific to intelligence.

Our model has enough parameters to approximately interpolate training data. This does not lead to overfitting, as can be seen in the results, however. This might be due to the double‐descent phenomenon (Belkin, Hsu, & Mitra, 2018). According to this theory, when model capacity surpasses the interpolation threshold, the solution space of the problem is enlarged, leading to flatter solutions on parameter space. We can hypothesize that even larger, more expressive, networks will not degrade performance, pointing toward possible improvements with increasing computational resources.

Abrol et al. (2021) demonstrates systematically that deep models benefit from representation learning, extracting more informative features from data than hand‐crafted ones. Previous benchmarks comparing deep models with traditional machine learning could be thus biased against deep models. Applying deep neural networks to lower level data, that is, timeseries instead of hand‐crafted features such as RSFC, can result in superior performance. We verified this in our comparison against a strong benchmark, based on penalized linear modeling.

Since RNNs are very flexible nonlinear models we cannot rule out the possibility that the RNNs could have learned a proxy measure of RSFC directly from data. RSFC is largely driven by a small number of high amplitude, small time‐scale, coactivity events (Esfahlani et al., 2020), thus also reflecting dynamically rich information.

Obtaining maximal performance was not our main objective. Instead, we aimed at providing a differentiable model relating RS‐fMRI activity to “g” and extracting relevant neuroscientific insights from it. The study of brain dynamics in relation to human intelligence warrants the use of models that use that information. In special, the leading linear modeling approaches are based on univariate filtering. Univariate filtering is nondifferentiable, precluding us from relying on gradients for the study of model reliance.

We demonstrated how one can decouple instant innovations from functional connectivity contributions using ZCA. It is possible to embed this knowledge into networks. Future works could then fuse models working on the functional‐connectivity domain and the time activity domain simultaneously. Such models could better differentiate the roles of dynamics in interindividual variations in cognition.

We successfully reproduced several findings from the literature pertaining to brain biology in the context of general intelligence. We built a model that predicts “g” from time‐distributed BOLD fMRI activity. This model attains slightly increased performance in this task, without filtering. Studying its reliance on different parts of input information allows us to retrieve neuroscientific insights. We also present a method based on propagating saliencies from data to derivative measures, such as functional connectivity. Using it, we disambiguate the contributions of instantaneous innovations to model reliance, for example. Combining an ablation‐based approach with a saliency based one allowed us to identify a set of regions that degrade performance significantly more than equally sized RSNs.

## CONFLICT OF INTEREST

The authors declare no potential conflict of interest.

## Data Availability

All data, imaging, demographical or behavioral, used is provided by the Human Connectome Project main study, HCP Young Adult. Data and details can be obtained at their site, pending approval. See https://www.humanconnectome.org/. Neural networks were implemented in Flux v0.9.0. See https://github.com/FluxML/Flux.jl/tree/v0.9.0. Additional code for analyses was implemented in Julia v1.3.0. See https://github.com/JuliaLang/julia/tree/v1.3.0. Code will be shared upon request.

## PROPAGATING DERIVATIVES TO NONSTANDARDIZED DATA

Implicitly, our model $N\left(X\right)$ includes standardization and BPF. For efficiency, we performed these operations *ante‐hoc*. When calculating saliencies, however, the model is agnostic to those operations. In special, saliencies are not, in principle, forbidden of inducing mean‐ and scale‐shifts, or to access forbidden frequencies below 0.008 Hz. The full model is, actually $N\left(S\left(H\left(X\right)\right)\right)$. $H\left(\cdot \right)$ represents BPF while $S\left(\cdot \right)$ represents temporal standardization. The ordering of the operations is not arbitrary, however. Since BPF does not preserve variance, it must be nested under standardization.

To retrieve meaningful saliencies we use the chain rule to arrive at the results shown in Equation (A1). We introduce the identities $H=H\left(X\right)$ and $S=S\left(H\right)$ to simplify notation.

(A1)
$$\frac{\partial N\left(S\right)}{\partial X}=\frac{\partial H}{\partial X}\frac{\partial S}{\partial H}\frac{\partial N\left(S\right)}{\partial S}$$

For any given region i we can express its timeseries as the column matrix $x_{i}=\left\{x_{\mathrm{it}}\right\}$. Its filtered version is $h_{i}=D^{T}{\mathrm{Dx}}_{i}$. D is the discrete Fourier transform (DFT) matrix with zeros for the elements of filtered frequencies. We can see from Equation (A2) that its derivatives are easy to compute.

(A2)
$$\frac{\partial h_{i}}{\partial x_{i}}=D^{T}D$$

The standardized version of $h_{i}$ is $s_{i}$, shown in Equation (A3).

(A3)
$$\left\{\begin{array}{l} s_{\mathrm{it}}=\sigma_{i}^{-1}\left(h_{\mathrm{it}}-\mu_{i}\right)\\ \mu_{i}=M^{-1}\sum_{t}^{M}h_{\mathrm{it}}=M^{-1}1^{T}h_{i}\\ \sigma_{i}=\sqrt{{\left(M-1\right)}^{-1}\sum_{t}^{M}{\left(h_{\mathrm{it}}-\mu_{i}\right)}^{2}}=\sqrt{{\left(M-1\right)}^{-1}\left(h_{i}^{T}h_{i}-{\left(1^{T}h_{i}\right)}^{2}\right)} \end{array}\right.$$

We can therefore express its partial derivatives regarding the elements in $h_{i}$.

(A4)
$$\frac{\partial s_{\mathrm{it}}}{\partial h_{\mathrm{ik}}}=\frac{\partial }{\partial h_{\mathrm{ik}}}\left(\frac{h_{\mathrm{it}}-\mu_{i}}{\sigma_{i}}\right)=\frac{1}{\sigma_{i}}\frac{\partial \left(h_{\mathrm{it}}-\mu_{i}\right)}{\partial h_{\mathrm{ik}}}-\frac{\left(h_{\mathrm{it}}-\mu_{i}\right)}{\sigma_{i}^{2}}\frac{\partial \sigma_{i}}{\partial h_{\mathrm{ik}}}$$

(A5)
$$\frac{\partial \left(h_{\mathrm{it}}-\mu_{i}\right)}{\partial h_{\mathrm{ik}}}=\delta_{\mathrm{ik}}-\frac{1}{M}$$

(A6)
$$\frac{\partial \sigma_{i}}{\partial h_{\mathrm{ik}}}=\frac{h_{\mathrm{ik}}-\mu_{i}}{\left(M-1\right)\sigma_{i}}$$

Combining Equations (A4), (A5), and (A6) results in Equation (A7).

(A7)
$$\frac{\partial s_{\mathrm{it}}}{\partial h_{\mathrm{ik}}}=\frac{\delta_{\mathrm{ik}}-M^{-1}}{\sigma_{i}}-\frac{\left(h_{\mathrm{it}}-\mu_{i}\right)\left(h_{\mathrm{ik}}-\mu_{i}\right)}{\left(M-1\right)\sigma_{i}^{3}}$$

These tensor derivatives can be flattened then recomposed into respective Jacobian matrices in Equation (A1).

## PROPAGATING GRADIENTS TO SVD COMPONENTS

The ZCA of a matrix $X={\mathrm{USV}}^{T}$ is given by $Z={\mathrm{UV}}^{T}$. Using the differential dZ it becomes simple to show that $dZ=d{\mathrm{UV}}^{T}+UdV^{T}$. Using the results obtained in (Townsend, 2016), dV and dU can be given in function of X and dX as shown in Equation (B1).

(B1)
$$\left\{\begin{array}{l} dU=U\left(F\circ \left(U^{T}d\mathrm{XVS}+{\mathrm{SV}}^{T}dX^{T}U\right)\right)+\left(I-{\mathrm{UU}}^{T}\right)d{\mathrm{XVS}}^{-1}\\ dV=V\left(F\circ \left(SU^{T}d\mathrm{XV}+V^{T}dX^{T}\mathrm{US}\right)\right)+\left(I-{\mathrm{VV}}^{T}\right)dX^{T}{\mathrm{US}}^{-1}\\ dS=I\circ \left(U^{T}d\mathrm{XV}\right) \end{array}\right.$$

The square matrix F is given in terms on S in Equation (B2).

(B2)
$$f_{i,j}=\left\{\begin{array}{l} \frac{1}{s_{j}^{2}-s_{i}^{2}}\;i\neq j\\ 0\;i=j \end{array}\right.$$

## References

[hbm25656-bib-0001] Abrol, A. , Fu, Z. , Salman, M. , Silva, R. , Du, Y. , Plis, S. , & Calhoun, V. (2020). Hype versus hope: Deep learning encodes more predictive and robust brain imaging representations than standard machine learning. *bioRxiv* (preprint). doi: 10.1101/2020.04.14.041582.

[hbm25656-bib-0002] Abrol, A. , Fu, Z. , Salman, M. , Silva, R. , Du, Y. , Plis, S. , & Calhoun, V. (2021). Deep learning encodes robust discriminative neuroimaging representations to outperform standard machine learning. Nature Communications, 12, 1–17. 10.1038/s41467-020-20655-6 PMC7806588 33441557

[hbm25656-bib-0003] Anticevic, A. , Cole, M. W. , Murray, J. D. , Corlett, P. R. , Wang, X.‐j. , & Krystal, J. H. (2012). The role of default network deactivation in cognition and disease. Trends in Cognitive Sciences, 16, 584–592. 10.1016/j.tics.2012.10.008 23142417 PMC3501603

[hbm25656-bib-0004] Barbey, A. K. (2018). Network neuroscience theory of human intelligence. Trends in Cognitive Sciences, 22, 8–20. 10.1016/j.tics.2017.10.001 29167088

[hbm25656-bib-0005] Belkin, M. , Hsu, D. , & Mitra, P. P. (2018). Overfitting or perfect fitting? Risk bounds for classification and regression rules that interpolate. Advances in Neural Information Processing Systems (NeurIPS), 31, 2300–2311. arXiv:1806.05161.

[hbm25656-bib-0006] Bezanson, J. , Edelman, A. , Karpinski, S. , & Shah, V. B. (2017). Julia: A fresh approach to numerical computing. SIAM Review, 59, 65–98. 10.1137/141000671 arXiv:1411.1607.

[hbm25656-bib-0007] Brookes, M. J. , Woolrich, M. , Luckhoo, H. , Price, D. , Hale, J. R. , Stephenson, M. C. , … Morris, P. G. (2011). Investigating the electrophysiological basis of resting state networks using magnetoencephalography. Proceedings of the National Academy of Sciences of the United States of America, 108, 16783–16788. 10.1073/pnas.1112685108 21930901 PMC3189080

[hbm25656-bib-0008] Calhoun, V. D. , & Adali, T. (2012). Multisubject independent component analysis of fMRI: A decade of intrinsic networks, default mode, and neurodiagnostic discovery. IEEE Reviews in Biomedical Engineering, 5, 60–73. 10.1109/RBME.2012.2211076 arXiv:15334406. 23231989 PMC4433055

[hbm25656-bib-0009] Calhoun, V. D. , Adali, T. , Pearlson, G. D. , & Pekar, J. J. (2001). A method for making group inferences from functional MRI data using independent component analysis. Human Brain Mapping, 14, 140–151. 10.1002/hbm.1048 11559959 PMC6871952

[hbm25656-bib-0010] Colom, R. , Karama, S. , Jung, R. E. , & Haier, R. J. (2010). Human intelligence and brain networks. Dialogues in Clinical Neuroscience, 12, 489–501. 10.31887/DCNS.2010.12.4/rcolom 21319494 PMC3181994

[hbm25656-bib-0011] Dizaji, A. S. , Vieira, B. H. , Khodaei, M.‐R. , Ashrafi, M. , Parham, E. , Hossein‐Zadeh, G.‐A. , … Soltanian‐Zadeh, H. (2021). Linking brain biology to intellectual endowment: A review on the associations between human intelligence and neuroimaging data. Basic and Clinical Neuroscience, 12, 1–28. 10.32598/bcn.12.1.574.1 33995924 PMC8114859

[hbm25656-bib-0012] Dubois, J. , Galdi, P. , Paul, L. K. , & Adolphs, R. (2018). A distributed brain network predicts general intelligence from resting‐state human neuroimaging data. Philosophical Transactions of the Royal Society B: Biological Sciences, 373, 20170284. 10.1098/rstb.2017.0284 PMC6107566 30104429

[hbm25656-bib-0013] Dvornek, N. C. , Ventola, P. , Pelphrey, K. A. , Duncan, J. S. (2017). Identifying autism from resting‐state fMRI using long short‐term memory networks. In Q. Wang, Y. Shi, & K. Suzuki (Eds.), *Machine learning in medical imaging*. Lecture Notes in Computer Science (pp. 362–370). Springer International Publishing. doi: 10.1007/978-3-319-67389-9_42 . PMC5669262 29104967

[hbm25656-bib-0014] Esfahlani, F. Z. , Jo, Y. , Faskowitz, J. , Byrge, L. , Kennedy, D. P. , Sporns, O. , & Betzel, R. F. (2020). High‐amplitude cofluctuations in cortical activity drive functional connectivity. Proceedings of the National Academy of Sciences of the United States of America, 117, 28393–28401. 10.1073/pnas.2005531117 33093200 PMC7668041

[hbm25656-bib-0015] Essen, D. C. V. , Smith, S. M. , Barch, D. M. , Behrens, T. E. , Yacoub, E. , & Ugurbil, K. (2013). The WU‐Minn human connectome project: An overview. NeuroImage, 80, 62–79. 10.1016/j.neuroimage.2013.05.041 arXiv:NIHMS150003. 23684880 PMC3724347

[hbm25656-bib-0016] Ferreira, L. K. , Regina, A. C. B. , Kovacevic, N. , Martin, M. D. G. M. , Santos, P. P. , Carneiro, C. D. G. , … Busatto, G. F. (2016). Aging effects on whole‐brain functional connectivity in adults free of cognitive and psychiatric disorders. Cerebral Cortex, 26, 3851–3865. 10.1093/cercor/bhv190 26315689

[hbm25656-bib-0017] Finn, E. S. , Shen, X. , Scheinost, D. , Rosenberg, M. D. , Huang, J. , Chun, M. M. , … Constable, R. T. (2015). Functional connectome fingerprinting: Identifying individuals using patterns of brain connectivity. Nature Neuroscience, 18, 1664–1671. 10.1038/nn.4135 arXiv:15334406. 26457551 PMC5008686

[hbm25656-bib-0018] Graves, A. , & Schmidhuber, J. (2005). Framewise phoneme classification with bidirectional LSTM and other neural network architectures. Neural Networks, 18, 602–610. 10.1016/j.neunet.2005.06.042 16112549

[hbm25656-bib-0019] He, T. , Kong, R. , Holmes, A. J. , Nguyen, M. , Sabuncu, M. R. , Eickhoff, S. B. , … Yeo, B. T. (2020). Deep neural networks and kernel regression achieve comparable accuracies for functional connectivity prediction of behavior and demographics. NeuroImage, 206, 116276. 10.1016/j.neuroimage.2019.116276 31610298 PMC6984975

[hbm25656-bib-0020] Hearne, L. J. , Mattingley, J. B. , & Cocchi, L. (2016). Functional brain networks related to individual differences in human intelligence at rest. Scientific Reports, 6, 32328. 10.1038/srep32328 27561736 PMC4999800

[hbm25656-bib-0021] Hochreiter, S. , & Schmidhuber, J. (1997). Long short‐term memory. Neural Computation, 9, 1735–1780. 10.1162/neco.1997.9.8.1735 9377276

[hbm25656-bib-0022] Innes, M. (2018). Flux: Elegant machine learning with Julia. Journal of Open Source Software, 3, 602. 10.21105/joss.00602

[hbm25656-bib-0023] Ito, T. , Kulkarni, K. R. , Schultz, D. H. , Mill, R. D. , Chen, R. H. , Solomyak, L. I. , & Cole, M. W. (2017). Cognitive task information is transferred between brain regions via resting‐state network topology. Nature Communications, 8, 1–13. 10.1038/s41467-017-01000-w PMC5715061 29044112

[hbm25656-bib-0024] Jensen, A. R. (1998). The Discovery of g. The g factor: The science of mental ability, Human Evolution, Behavior, and Intelligence, (1st ed., pp. 18–44). Westport, Connecticut and London: Praeger. 10.1007/s13398-014-0173-7.2

[hbm25656-bib-0025] Jung, R. E. , & Haier, R. J. (2007). The Parieto‐frontal integration theory (P‐FIT) of intelligence: Converging neuroimaging evidence. Behavioral and Brain Sciences, 30, 135–154. 10.1017/S0140525X07001185 17655784

[hbm25656-bib-0026] Kingma, D.P. , Ba, J. (2014). Adam: A method for stochastic optimization, *arXiv Preprint*. pp. 1–15. arXiv:1412.6980.

[hbm25656-bib-0027] Kohavi, R. , & Edu, S. (1993). A study of cross‐validation and bootstrap for accuracy estimation and model selection. Proceedings of the 14th International Joint Conference on Artificial Intelligence, 2, 1137–1143.

[hbm25656-bib-0028] Krizhevsky, A. (2009). Learning multiple layers of features from tiny images. Retrieved from https://www.cs.toronto.edu/~kriz/learning-features-2009-TR.pdf

[hbm25656-bib-0029] Krizhevsky, A. , Sutskever, I. , & Hinton, G. E. (2012). ImageNet classification with deep convolutional neural networks. Advances in Neural Information Processing Systems, 2, 1097–1105.

[hbm25656-bib-0030] Krogh, A. , & Hertz, J. A. (1992). A simple weight decay can improve generalization. Advances in Neural Information Processing Systems, 4, 950–957.

[hbm25656-bib-0031] Loshchilov, I. , Hutter, F. (2019). *Decoupled weight decay regularization*. 7th International Conference on Learning Representations, ICLR 2019. arXiv:1711.05101.

[hbm25656-bib-0032] Luders, E. , Narr, K. L. , Thompson, P. M. , & Toga, A. W. (2009). Neuroanatomical correlates of intelligence. Intelligence, 37, 156–163. 10.1016/j.intell.2008.07.002 20160919 PMC2770698

[hbm25656-bib-0033] McDaniel, M. A. (2005). Big‐brained people are smarter: A meta‐analysis of the relationship between in vivo brain volume and intelligence. Intelligence, 33, 337–346. 10.1016/j.intell.2004.11.005

[hbm25656-bib-0034] Molnar, C. (2019). Model‐agnostic methods. In Interpretable machine learning: A guide for making black box models explainable. Munich, Germany. Retrieved from https://christophm.github.io/interpretable-ml-book/

[hbm25656-bib-0035] Pamplona, G. S. P. , Santos Neto, G. S. , Rosset, S. R. E. , Rogers, B. P. , & Salmon, C. E. G. (2015). Analyzing the association between functional connectivity of the brain and intellectual performance. Frontiers in Human Neuroscience, 9, 61. 10.3389/fnhum.2015.00061 25713528 PMC4322636

[hbm25656-bib-0036] Ritchie, S. J. , Booth, T. , Hernández, M. D. C. V. , Corley, J. , Maniega, S. M. , Gow, A. J. , … Deary, I. J. (2015). Beyond a bigger brain: Multivariable structural brain imaging and intelligence. Intelligence, 51, 47–56. 10.1016/j.intell.2015.05.001 26240470 PMC4518535

[hbm25656-bib-0037] Spearman, C. (1904). "general intelligence," objectively determined and measured. The American Journal of Psychology, 15, 201. 10.2307/1412107 14717630

[hbm25656-bib-0038] Sridharan, D. , Levitin, D. J. , & Menon, V. (2008). A critical role for the right fronto‐insular cortex in switching between central‐executive and default‐mode networks. Proceedings of the National Academy of Sciences of the United States of America, 105, 12569–12574. 10.1073/pnas.0800005105 18723676 PMC2527952

[hbm25656-bib-0039] Sui, J. , Liu, M. X. , Lee, J. H. , Zhang, J. , & Calhoun, V. (2020). Deep learning methods and applications in neuroimaging. Journal of Neuroscience Methods, 339, 108718. 10.1016/j.jneumeth.2020.108718 32272117 PMC9245029

[hbm25656-bib-0040] Sun, F. T. , Miller, L. M. , & D'Esposito, M. (2004). Measuring interregional functional connectivity using coherence and partial coherence analyses of fMRI data. NeuroImage, 21, 647–658. 10.1016/j.neuroimage.2003.09.056 14980567

[hbm25656-bib-0041] Thurstone, L. L. (1940). Current issues in factor analysis. Psychological Bulletin, 37, 189–236.

[hbm25656-bib-0042] Townsend, J. (2016). Differentiating the singular value decomposition. Available from https://j-towns.github.io/papers/svd-derivative.pdf.

[hbm25656-bib-0043] Williams, R. J. , & Peng, J. (1990). An efficient gradient‐based algorithm for on‐line training of recurrent network trajectories. Neural Computation, 2, 490–501. 10.1162/neco.1990.2.4.490

[hbm25656-bib-0044] Yekutieli, D. , & Benjamini, Y. (1999). Resampling‐based false discovery rate controlling multiple test procedures for correlated test statistics. Journal of Statistical Planning and Inference, 82, 171–196. 10.1016/s0378-3758(99)00041-5

[hbm25656-bib-0045] Yeo, B. T. T. , Krienen, F. M. , Sepulcre, J. , Sabuncu, M. R. , Lashkari, D. , Hollinshead, M. , … Buckner, R. L. (2011). The organization of the human cerebral cortex estimated by intrinsic functional connectivity. Journal of Neurophysiology, 106, 1125–1165. 10.1152/jn.00338.2011 21653723 PMC3174820

